# Towards manufacturing high uniformity polysilicon circuits through TFT contact barrier engineering

**DOI:** 10.1038/s41598-018-35577-z

**Published:** 2018-12-03

**Authors:** Radu A. Sporea, Luke J. Wheeler, Vlad Stolojan, S. Ravi P. Silva

**Affiliations:** Advanced Technology Institute, Department of Electrical and Electronic Engineering, University of Surrey, Guildford, Surrey, GU2 7XH UK

## Abstract

The predicted 50 billion devices connected to the Internet of Things by 2020 has renewed interest in polysilicon technology for high performance new sensing and control circuits, in addition to traditional display usage. Yet, the polycrystalline nature of the material presents significant challenges when used in transistors with strongly scaled channel lengths due to non-uniformity in device performance. For these new applications to materialize as viable products, uniform electrical characteristics on large areas will be essential. Here, we report on the effect of deliberately engineered potential barrier at the source of polysilicon thin-film transistors, yielding highly-uniform on-current (<8% device-to-device, accounting for material, as well as substantial geometrical, variations). The contact-controlled architecture of these transistors significantly reduces kink effect and produces high intrinsic gain over a wide range of drain voltage (2–20 V). TCAD simulations associate critical grain boundary position and the two current injection mechanisms in this type of device, showing that, for the geometry considered, the most unfavorable location is ~150 nm inside the source area. At this point, grain boundary contributes to increasing the resistance of the source pinch-off region, reducing the current injection from the bulk of the source area. Nevertheless, the effect is marginal, and the probability of a grain boundary existing at this position is low. This new understanding is instrumental in the design of new signal conversion and gain circuits for flexible and low-power sensors, without the need for complex compensation methods.

## Introduction

Recent advances into polysilicon thin-film transistors^1–3^ (TFTs) are moving beyond^4–12^ their traditional display^13–16^ applications. These steps will contribute to realizing practical, low-power, low-cost products in emerging sectors, including: Internet-of-Things, wearable, medical, personal entertainment. For these transitions to translate into viable commercial products, achieving uniformity of electrical characteristics over a large area will be critical in ensuring high yield alongside superior electrical performance. Polysilicon circuits are prime candidates as the platform of choice for many of these applications, based on technology maturity, as well as performance resulting from high charge carrier mobility^17–28^. However, the polycrystalline nature of the material induces device-to-device variability due to the arbitrary number and position of grain boundaries in the transistor channel. This deleterious effect may be more pronounced when the material is purposely engineered for very high carrier mobility via large grains; the presence of a grain boundary in a transistor’s active region results in a significant lowering of the effective mobility, and of drain current. Mitigating strategies for compensating the effects of random grain boundary positions include current-mode driving schemes or compensation circuitry^17,29–33^. These solutions increase design time, power consumption, and circuit complexity, affecting yield and application viability. Large and single grain techniques have been investigated, with advantages in current uniformity, but at the expense of integration density and fabrication complexity.

We propose and investigate a polysilicon TFT structure in which current is controlled by a intentionally-engineered potential barrier at the source, which reduces the adverse effect of a grain boundary in the active area of the device. This device, known as the source-gated transistor (SGT)^3,34^, has shown very low saturation voltage^35–37^ and dramatically reduced kink effect^38–43^ without the need of special implants or additional processing steps^37,44^. This device concept has valuable characteristics for both analog^45–47^ and digital^48^ circuits. Schottky barrier TFTs using the same control principle have been demonstrated to provide a route to achieving similar operating advantages in many other material systems^49–59^.

## Source-Gated Transistor Construction and Operation

**Figure 1:**
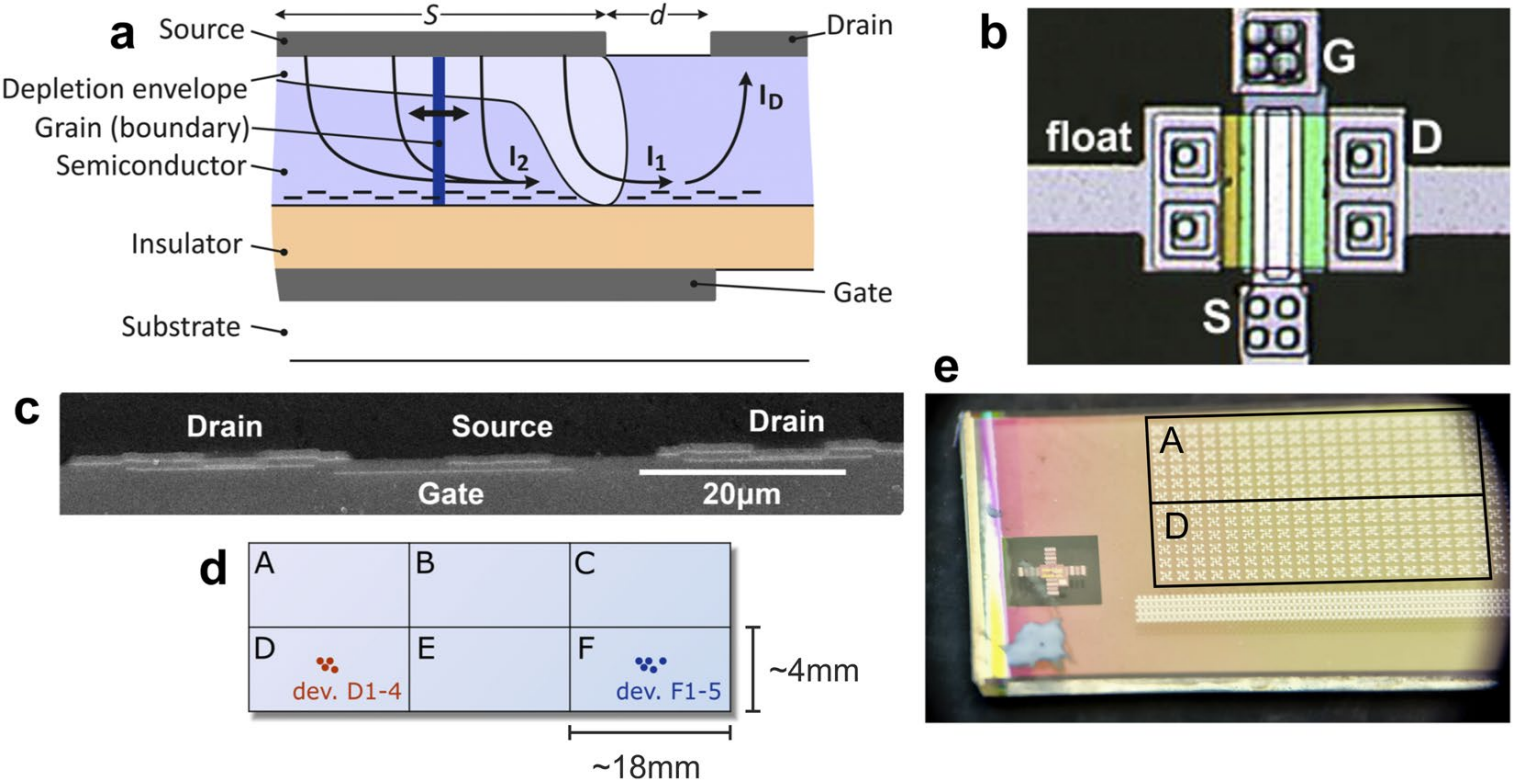
(**a**) Schematic cross-section of a bottom-gate, top-contact, *n-type*, source-gated transistor. The source comprises a potential barrier. Positive V_G_ accumulates charge at the insulator interface. Positive V_D_ reverse biases the source, resulting in full depletion of the semiconductor at the edge of the source – source pinch-off. Injection occurs in two distinct Modes: I_1_ is the current injected in the pinch-off region and modulated by (gate) electric field; I_2_ is injected from the bulk of the source as a result of the potential difference across the semiconductor layer^60^. In simulations, a single grain boundary (dark blue) was swept laterally to study its influence on electrical characteristics. (**b**) Micrograph of a polysilicon source-gated transistor (SGT); W = 50 µm; d = 10 µm; (**c**) SEM cross-section of a polysilicon SGT; d = 10 µm; (**d**) Device arrays repeat six times on the substrate, and the measured devices are clustered in 2 mm^2^ regions in their respective repeating arrays; (**e**) close-up photograph of the SGT arrays fabricated on glass via photolithography.

In normal operation, the Schottky-source SGT relies on injection through the reverse-biased source contact barrier to regulate current (Fig. 1a). The semiconductor pinches off at significantly lower drain voltage than in conventional TFTs, at the edge of the source electrode closest to the drain. Effects include early saturation of drain current and flat output characteristics in saturation over a wide range of drain voltages^37,44,53,60^. Two distinct current injection mechanisms contribute to overall drain current in this type of device. Firstly, the pinch-off region at the edge of the source allows the gate-induced electric field to reach the metal-semiconductor contact, lowering the effective source barrier height in this region, to modulate its reverse current (I_1_, Fig. 1a)^61^. This represents Mode I of SGT operation^55,60^, and was the first mechanism identified when SGT devices were originally proposed.

Secondly, injection from the bulk of the source electrode is defined as Mode II (I_2_, Fig. 1a). The current injected in this mode is practically ohmic in nature and dependent on the potential along the accumulation layer under the source^55,60^. The drain current is the sum of I_1_ and I_2_ currents, and has characteristics determined by the dominant current of the two^62,63^. For practical reasons, including improved saturation behaviour and lower temperature coefficient of the drain current, it is usually advantageous to design the device such that I_2_ dominates, requiring that the gate and source electrodes overlap (Fig. 1a), to create the necessary accumulation layer under the source. SGT-like behavior can be obtained from devices with coplanar contacts, however these devices would only achieve flat saturated characteristics by including lateral field-relief structures^44^.

Here, we report on polysilicon SGTs with high drain current uniformity which results from the current control mechanism. TCAD simulations permit systematic analysis of the device physics and confirm that the current does not depend on the grain boundary position, save for a precise location within the source area relative to the depletion region at the edge of the source electrode. Moreover, these results confirm the suitability of the SGT device structure as a viable element for robust circuit design in high-performance polycrystalline semiconductor technologies.

### Polysilicon source-gated transistors

**Figure 2:**
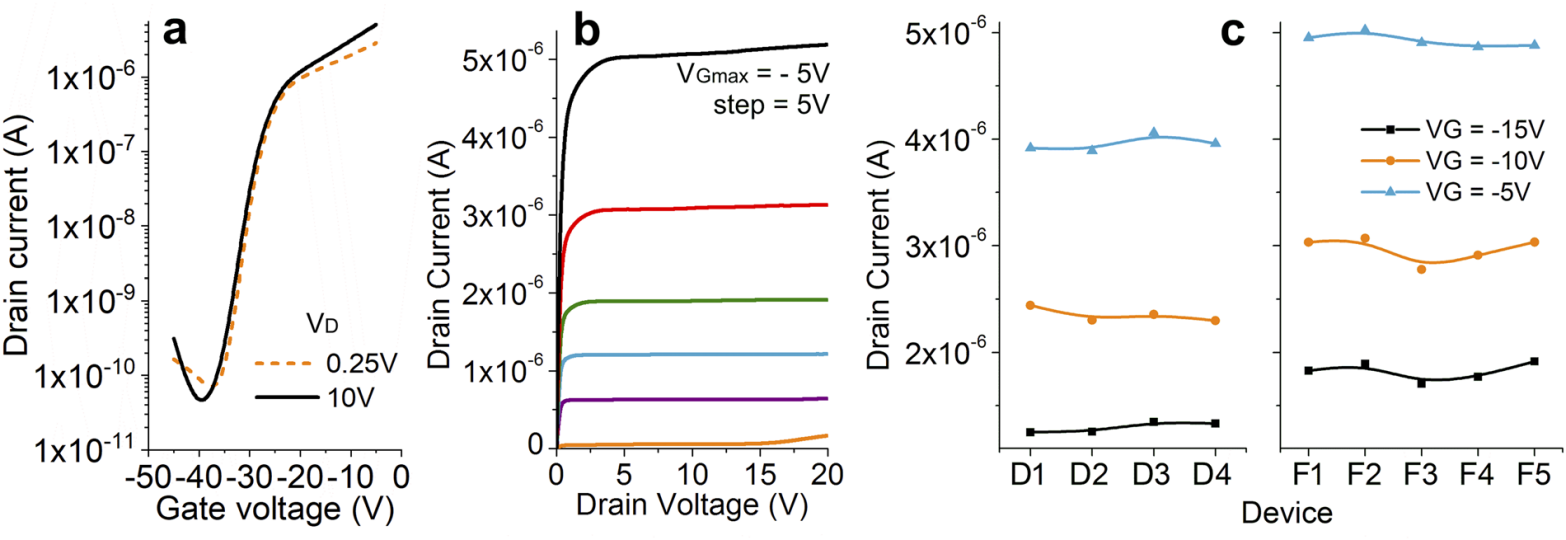
(**a**) Measured transfer characteristics for an *n-type* SGT with W = 50 µm; d = 2 µm; source-gate overlap S = 8 µm for two V_D_ values; (**b**) Measured output curves for the same device. Typical low saturation voltage and low V_D_ dependence of drain current are observed. Threshold voltage can be tuned by bulk doping^37^. (**c**) Measured variation in drain current for nine devices as identified in Fig. 1d. Consistency is observed despite material non-uniformities, position on the substrate, and geometrical variations. W = 50 µm; d = 2, 4, 10 µm; source-gate overlap S = 4, 6, 8 µm.

Devices were fabricated in a bottom-gate, self-aligned-drain process (Fig. 1b–e). The transfer characteristic of a representative device are shown in Fig. 2a. Measurements confirm typical SGT behavior: low saturation voltage due to source-end channel pinch-off and flat output characteristics to high V_D_ with negligible kink effect (Fig. 2b). The curves shown belong to a device with large negative threshold, but transistors with near-zero or positive thresholds behave similarly^37,64^. The vertical geometry of these devices was not optimized for low-voltage operation, and significant reduction in gate voltage can be obtained by reducing the gate insulator thickness. The subthreshold characteristic of these types of devices is largely dominated by the accumulation channel and behavior is practically identical to a conventional FET. The off-state current is determined largely by the injection across the source contact, and these devices show I_off_ < 10^−11^A for all geometries. In Fig. 2c, the drain current at three operating points is plotted for several devices across the substrate, showing less than 8% device-to-device variation in the same area (D or F, see Fig. 1d). It is important to note that this variation takes into account local material variability, as well as large geometrical variations (source-drain gap, source-gate overlap area), which the SGT can accommodate well. We attribute the change in current between areas of the wafer to registration errors due to thermal expansion of the substrate during processing for this batch of devices. This has been documented previously (e.g. ref.^37^) and is largely linked with the alignment of metal field-relief structures.

### TCAD analysis and grain boundary effects

**Figure 3:**
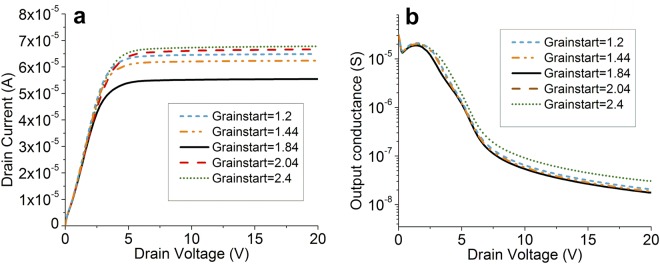
(**a**) Simulated output curve for different horizontal locations of the grain boundary. The source electrode ends at *x* = 2 µm. Current is unaffected, save for a single area adjacent to the source depletion region (Fig. 1a). ϕ_m_ = 4.47 eV; V_G_ = 15 V. (**b**) Output conductance plot extracted from (**a**), showing consistency regardless of the position of the grain boundary. Intrinsic gain, and implicitly circuit behavior, remain unaffected.

We have performed TCAD simulations on a 2D model of the structure using Silvaco Atlas, considering a 3 µm source-drain gap, a 2 µm source length, and assuming the semiconductor as crystalline. We can then characterize the effect of introducing a single grain boundary in various areas of the device, according to its location relative to the transistor’s functional regions. We perform the simulation with a single boundary in order to illustrate specific device behavior related to different gran boundary position. The presence of a single grain boundary in the bulk of the source (x < 1.5 µm), in the channel (2 µm< x <5 µm), or in the drain region ( x >5 µm, not shown) does not affect drain current distinguishably (Fig. 3a). This is attributed to the current control mechanism, as will be discussed below. However, there is a drop in total drain current when a grain boundary is located just inside the source region (x = 1.84 µm). Significantly, the output conductance, g_d_ = dI_D_/dV_D_ shows only modest variation with grain boundary position (Fig. 3b), which is linked to current magnitude (lower g_d_ for lower I_D_). From a circuit perspective, this is encouraging: the simultaneous small reduction in transconductance, g_m_ = dI_D_/dV_G_ with drain current, causes the device’s intrinsic gain, A_V_ = g_m_/g_d_, to remain approximately constant, regardless of material properties.

Below we detail the insights into device operation provided by TCAD. The results allow us to justify the critical grain boundary position, namely x = 1.84 µm in the structures considered, and to explain the robustness of the device’s behavior for all other grain boundary locations.

**Figure 4:**
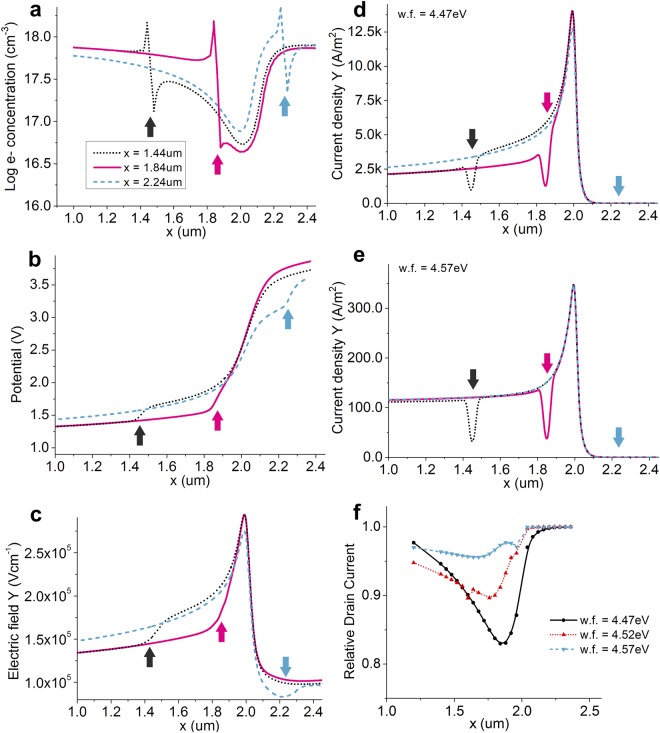
(**a**) Simulated carrier concentration at the semiconductor-insulator interface. Arrows indicate the position of the grain boundary. The critical position of the grain boundary is just inside the source contact (*x* < 2 µm), effectively widening the pinch-off region and increasing its total resistance (magenta arrow). Other positions (black and blue arrow) do not affect the pinch-off region, and hence, drain current. ϕ_m_ = 4.47 eV; V_G_ = 15 V; V_D_ = 20 V. (**b**) Simulated potential at the semiconductor-insulator interface. A large potential drop at the source edge is observed when a grain boundary lay just inside the source region (magenta arrow). This reduces the potential developed vertically across the semiconductor in the bulk of the source, resulting in lower injection from this area, and lower total drain current, when compared to cases when the grain boundary is either side of the source edge. ϕ_m_ = 4.47 eV; V_G_ = 15 V; V_D_ = 20 V. (**c**) Simulated normal electric field at the semiconductor-insulator interface, showing a no significant change in the maximum electric field in the pinch-off region with grain boundary position. This results in constant injection levels form the Mode I injection mechanism^60^, regardless of the grain boundary location. ϕ_m_ = 4.47 eV; V_G_ = 15 V; V_D_ = 20 V. Current density in the Y direction at the source contact is plotted for (**d**) ϕ_m_ = 4.47 eV and (**e**) ϕ_m_ = 4.57 eV, illustrating the reduced injection at the grain boundary, and the lower injection from the bulk due to potential drop at the grain boundary. (**f**) Drain current dependence on grain boundary position and source contact work function.

First, we will consider the current injected from the bulk of the source region, I_2_. Figure 4a illustrates the electron concentration at the semiconductor-insulator interface for three positions of the grain boundary. The pinch-off region at the edge of the source (x = 2 µm) plays an important role in controlling the current^34,37,55,57,60^. Its shape is unaffected by grain boundaries within the source (x < 1.5 µm) or in the channel region (2 µm < × < 3 µm), and drain current does not change. However, a grain boundary situated just inside the source (x = 1.84 µm in Fig. 4a) lowers the drain current perceptibly, as seen in Fig. 3a. Its presence effectively widens the depletion region, increasing its total resistance. Current injected from the bulk of the source (I_2_ in Fig. 1e) traverses this region laterally at the semiconductor-insulator interface, and a larger potential drop is observed at the edge of the source (Fig. 4b, magenta arrow) when compared to any other grain boundary location. This reduces the potential in the accumulation layer underneath the source contact (e.g. at x = 1.6 µm in Fig. 4b). The magnitude of current I_2_ is dictated by the potential drop between the source electrode and the accumulation layer in the source area (SGT Operating Mode II^55,60^), and thus a more resistive pinch-off region, caused by the existence of the grain boundary in its vicinity, results in lower I_2_ current. Figure 4d shows the density of electron current injected at each point along the source. The presence of the grain boundary further lowers I_2_ owing to the reduced injection in that region of the semiconductor. When the grain boundary is located deep in the bulk of the source, both effects are minimized. This is due to the diminishing contribution to I_2_ from regions farther away from the edge of the source, resulting from potential drops in the accumulation layer. Further, a grain boundary located in the source-drain gap has no influence over potential under the source, and, as such, a grain boundary in the transistor channel does not affect drain current.

The total drain current includes contributions from the edge of the source (I_1_, reverse saturation current, modulated by electric field; Mode I)^60^, as well as from the bulk of the source (I_2_, ohmic, Mode II). If I_2_ dominates, the potential drop at the edge of the source attributed to widening of the depletion region would account for the lowering of drain current when a grain boundary is present just inside the source area.

Next, we analyze the influence of grain boundary position on the behavior of the I_1_ component of drain current by probing the means of its modulation, namely electric field in the region in which I_1_ originates^60^. Figure 4c shows the normal component of the electric field at the metal-semiconductor interface. The electric field magnitude in the pinch-off region (approx. x = 2 ± 0.05 µm) does not change significantly with grain boundary position. The modest dependence seen is related to the reduced potential drop across the depletion region when a grain boundary exists just inside the source-drain gap (Fig. 4b, blue dashed curve and blue arrow). Overall, this confirms a low dependence of I_1_ on grain boundary position. Moreover, I_1_ generally represents a comparatively small proportion of total drain current, especially in practical implementations where source-length (or source-drain overlap) is likely to be higher than S = 2 μm considered here.

We can conclude that the first order effect of the presence of a grain boundary is the change in I_2_ due to reduced potential in the accumulation layer under the source.

The device width is likely to be larger than the source-drain gap in usual applications, and as such, multiple grain boundaries are likely to be comprised in the direction (*y*) perpendicular to current flow. While this study does not directly address this effect, considering the usual grain formation and the physics of the device, it is safe to assume that, to a first order, the presence of grain boundaries in the *y* direction will have minimal impact, and their effect is easier to average than that of those in the *x* direction.

The most detrimental location of the grain boundary (here *x* = 1.84 um, equivalent to 160 nm into the source region) will vary slightly with device geometry. It is safe to assume that it will be in a position which maximally increases the total depletion width at the source. Since the depletion width is on the order of the semiconductor thickness, accounting for two-dimensional electric field distribution in this area of the device, it is plausible to assume that the critical grain boundary is within 50–200 nm from the edge of the source for practical polysilicon layer thickness. The generally-applicable conclusion, however, is that the presence of a grain boundary is only significantly detrimental if located only in a comparatively small region compared to the device dimensions in the *x* direction, therefore the probability of encountering a grain boundary at the critical position is low. Even in the worst-case scenario, the current reduction is modest, and likely to be tolerated by most applications.

### Barrier height dependent effects

Finally, we investigate the role of the source barrier height in the uniformity of drain current, linking the discussion to the position of the grain boundary. Barrier height will play a role in controlling I_1_ and I_2_ in different ways: I_1_ is exponentially dependent on φ_B_, as it represents the reverse saturation current of the barrier under an applied electric field; I_2_ only has a slight dependence on source barrier, being chiefly controlled by semiconductor layer resistance and potential drop across it^55,60^. Thus far, a comparatively low barrier height, φ_B_ (modelled here by the low source metal work function, φ_m_ = 4.47), has been used on account of the high on-current, transconductance and switching speed attainable. Practical studies of effective barrier heights and their behavior under bias have been conducted elsewhere^37^.

Increasing the barrier height (Fig. 4e) has the effect of lowering the impact of the presence of a grain boundary in the source region. A higher barrier increases the contribution of I_2_ to total drain current at the expense of I_1_^60^. Since I_1_ does not depend (or weakly depends) on grain boundary location, drain current is less affected in devices with higher source barriers. Concentrating on the behavior of I_2_, for high source barriers, the current injected from the bulk of the source reduces, and the potential drop at the grain boundary is also reduced. This lowers the variation of potential in the accumulation layer due to the presence of the grain boundary, in turn making I_2_ more resilient to grain boundary position. The only persistent effect at increased barrier height is the dip in injection current density at the grain boundary itself, with a modest influence on I_2_ magnitude. However, this latter behavior has similar contribution regardless of the grain boundary position, in contrast to the low-barrier case. We attribute this difference to the relatively low potential drop along the accumulation layer under the source when the barrier is high and current is low^60^.

This behavior is also synthesized in Fig. 4f, which plots the value of drain current as a proportion of that achieved in the absence of a grain boundary, versus source contact work function and grain boundary position. Low barriers lead to larger drain current variations when a grain boundary is present in the source region. For higher barriers, the drain current reduction is more consistent regardless of grain boundary position under the source. While higher barriers appear to lead to more uniform results, other effects such as operating speed, transconductance, current capability, drain field dependence, robustness against geometrical variability, etc. need to be considered, as required by the application.

## Conclusion

In summary, we have presented polysilicon thin-film transistors in which a Schottky source barrier provides the mechanism for current control by allowing full depletion of the semiconductor layer at the edge of the source (source-gated transistors - SGTs). The measured devices show minimal kink effect, low saturation voltage, and high device-to-device current uniformity.

The low dependence of current on the semiconductor’s crystalline structure results from the means of current control, and was studied via TCAD. The sole position of a grain boundary which causes a notable change in drain current is just inside the source depletion region, and represents a proportionally small fraction of total device size. Thus, the probability of a grain boundary being present in the critical region is low, especially for large grain sizes (e.g. >1 μm). Moreover, the absolute worst-case scenario identified yielded a 18% lower drain current, with <8% measured in devices with both material and geometrical differences.

These properties recommend polysilicon SGTs for high-gain, energy-efficient circuits with repeatable performance, and should provide a versatile design element at a time when polycrystalline semiconductors are receiving renewed worldwide interest.

## Methods

### Polysilicon transistor fabrication

Self-aligned, bottom gate, staggered electrode source-gated transistors (Fig. 1) were fabricated on glass substrates via photolithography. The process, described fully in ref.^37^, uses a thick gate dielectric stack (200 nm SiNx and 200 nm SiO2 by PECVD) which is largely responsible for the comparatively high gate voltages required^37^. 40 nm a-Si:H was deposited 40 nm and baked at 450 °C; BF2 or P were used as threshold-tuning bulk doping. The drain ohmic region was self-aligned to the gate via back exposure and P implantation. The semiconductor layer was then crystallized with an excimer laser and islands were defined by etching. BF2 or P implants were made through a SiO2 window to modify the source contact energy profile, and annealed at 550 °C. Cr/Al/Ti were used to form the Schottky (source) and ohmic (drain) contacts; only the right-side drain was probed (Fig. 1b) for this study. Devices with combinations of width (W), channel length (d), and source-gate overlap (S) were made in repeating arrays (Fig. 1d).

### TCAD modelling and simulation

Silvaco Atlas version 5.18.3.R was used for modelling and 2D simulation of a typical staggered-electrode SGT structure (Fig. 1a). To study the influence of material crystallinity, a single grain boundary (modelled as a 40 nm-long a:Si region with the default Silvaco Atlas parameters: defects nta = 1.e21 ntd = 1.e21 wta = 0.033 wtd = 0.049 nga = 4.5e15 ngd = 4.5e15 ega = 0.62 egd = 0.78 wga = 0.15 wgd = 0.15 sigtae = 1.e-17 sigtah = 1.e-15 sigtde = 1.e-15 sigtdh = 1.e-17 siggae = 2.e-16 siggah = 2.e-15 siggde = 2.e-15 siggdh = 2.e-16; material region = 2 mun = 20 mup = 1.5 nc300 = 2.5e20 nv300 = 2.5e20 eg300 = 1.9) was swept horizontally across a crystalline silicon active layer (again modelled with the default Altas parameters) in 20 nm steps.

Other geometrical parameters included: 200 nm SiO2 gate dielectric thickness, channel length d = 3 µm, source-gate overlap S = 2 µm. Active layer meshing was kept uniform at 10 nm vertically, and 20 nm horizontally.

The Schottky contact model was enabled for the source electrode, with surface recombination, and field-induced barrier lowering parameter α = 4 nm. The work function of the source metal was set to ϕ_m_ = 4.47 eV, and varied for the barrier height study.

## Data Availability

Data are available on request from the corresponding author.
